# Arterial resection and divestment in pancreatic cancer surgery in the era of multidisciplinary treatment: decadal comparative study

**DOI:** 10.1093/bjsopen/zraf026

**Published:** 2025-04-17

**Authors:** Yuki Hirose, Atsushi Oba, Yosuke Inoue, Aya Maekawa, Kosuke Kobayashi, Kojiro Omiya, Atsushi Takahashi, Yoshihiro Ono, Takafumi Sato, Hiromichi Ito, Takafumi Mie, Takashi Sasaki, Masato Ozaka, Naoki Sasahira, Toshifumi Wakai, Yu Takahashi

**Affiliations:** Division of Hepatobiliary and Pancreatic Surgery, Cancer Institute Hospital, Japanese Foundation for Cancer Research, Tokyo, Japan; Division of Digestive and General Surgery, Niigata University Graduate School of Medical and Dental Sciences, Niigata, Japan; Division of Hepatobiliary and Pancreatic Surgery, Cancer Institute Hospital, Japanese Foundation for Cancer Research, Tokyo, Japan; Division of Hepatobiliary and Pancreatic Surgery, Cancer Institute Hospital, Japanese Foundation for Cancer Research, Tokyo, Japan; Division of Hepatobiliary and Pancreatic Surgery, Cancer Institute Hospital, Japanese Foundation for Cancer Research, Tokyo, Japan; Division of Hepatobiliary and Pancreatic Surgery, Cancer Institute Hospital, Japanese Foundation for Cancer Research, Tokyo, Japan; Division of Hepatobiliary and Pancreatic Surgery, Cancer Institute Hospital, Japanese Foundation for Cancer Research, Tokyo, Japan; Division of Hepatobiliary and Pancreatic Surgery, Cancer Institute Hospital, Japanese Foundation for Cancer Research, Tokyo, Japan; Division of Hepatobiliary and Pancreatic Surgery, Cancer Institute Hospital, Japanese Foundation for Cancer Research, Tokyo, Japan; Division of Hepatobiliary and Pancreatic Surgery, Cancer Institute Hospital, Japanese Foundation for Cancer Research, Tokyo, Japan; Division of Hepatobiliary and Pancreatic Surgery, Cancer Institute Hospital, Japanese Foundation for Cancer Research, Tokyo, Japan; Department of Hepato-Biliary-Pancreatic Medicine, Cancer Institute Hospital, Japanese Foundation for Cancer Research, Tokyo, Japan; Department of Hepato-Biliary-Pancreatic Medicine, Cancer Institute Hospital, Japanese Foundation for Cancer Research, Tokyo, Japan; Department of Hepato-Biliary-Pancreatic Medicine, Cancer Institute Hospital, Japanese Foundation for Cancer Research, Tokyo, Japan; Department of Hepato-Biliary-Pancreatic Medicine, Cancer Institute Hospital, Japanese Foundation for Cancer Research, Tokyo, Japan; Division of Digestive and General Surgery, Niigata University Graduate School of Medical and Dental Sciences, Niigata, Japan; Division of Hepatobiliary and Pancreatic Surgery, Cancer Institute Hospital, Japanese Foundation for Cancer Research, Tokyo, Japan

## Abstract

**Background:**

The aim of this study was to investigate the feasibility and effectiveness of pancreatectomy with arterial resection/divestment for pancreatic cancer with arterial involvement in the modern era of multidisciplinary treatment.

**Methods:**

Patients who underwent pancreatectomy with arterial resection/pancreatectomy with arterial divestment for pancreatic cancer with arterial involvement from 2010 to 2021 were retrospectively analysed, and outcomes were compared between the former (2010–2015) and latter interval (2016–2021). Survivals were compared by univariable and multivariable analyses.

**Results:**

Among 203 patients included, 76 underwent pancreatectomy with arterial resection and 127 underwent pancreatectomy with arterial divestment. Compared with the former interval, more patients received preoperative chemotherapy (26.6% (*n* = 21) *versus* 95% (*n* = 118), *P* < 0.001), and underwent pancreatectomy with arterial resection (30.4% (*n* = 24) *versus* 41.9% (*n* = 52), *P* = 0.287) in the latter interval. The major morbidity rate and pancreatic fistula decreased in the latter interval (major morbidity rate: *P* = 0.040; pancreatic fistula: *P* = 0.006), even among patients undergoing pancreatectomy with arterial resection (major morbidity rate: *P* = 0.013; pancreatic fistula: *P* < 0.001). Patients in the latter interval had better overall survival (26.0 *versus* 48.2 months, *P* = 0.001), even among patients undergoing pancreatectomy with arterial resection (22.0 *versus* 45.1 months, *P* = 0.076).

**Conclusions:**

Within the context of modern multidisciplinary treatment, radical resection including arterial resection should be justified for patients with pancreatic cancer with arterial involvement, considering the acceptable perioperative risk and prolonged survival.

## Teaser

Compared with the former interval (2010–2015), the postoperative morbidity rate and early recurrence decreased, and overall survival and postrecurrence survival prolonged in the latter interval (2016–2021) for pancreatic cancer patients with arterial involvement undergoing pancreatectomy including arterial resection. Within the context of modern multidisciplinary treatment, radical resection including arterial resection can be justified for pancreatic cancer patients with arterial involvement at expert centres, considering the acceptable perioperative risk and the potential for prolonged survival.

## Introduction

Pancreatectomy with major arterial resection (PAR) for pancreatic cancer remains controversial. In 2011, a meta-analysis revealed a significantly high perioperative morbidity rate of 53% and mortality rate of 11.3%, along with diminished oncological survival^1^. Since effective multi-agent chemotherapy regimens including fluorouracil plus leucovorin plus irinotecan plus oxaliplatin (FOLFIRINOX) and gemcitabine plus nab-paclitaxel (GNP) emerged^2,3^ and have been gradually integrated into neoadjuvant/induction chemotherapy for borderline resectable pancreatic cancer (BRPC)/locally advanced pancreatic cancer (LAPC), increasing evidence has accumulated, demonstrating convincing short- and long-term outcomes following PAR for pancreatic cancer^4–9^. Studies exploring the outcomes of PAR included patients predating the application of these multi-agent regimens^5–7^. It should be necessary to assess the role of invasive surgical interventions in the era of modern multidisciplinary treatment.

The present study evaluated the short- and long-term outcomes of patients with pancreatic cancer with arterial involvement undergoing pancreatectomy with arterial divestment (PAD)/PAR, especially those during the era of modern multidisciplinary treatment in a high-volume pancreas centre. By conducting a decadal comparison, the factors that contribute to the improvement of long-term outcomes were explored.

## Methods

### Patient selection

This study analysed consecutive patients undergoing PAR/PAD for BRPC/LAPC with arterial involvement from 2010 to 2021 at the Cancer Institute Hospital, Tokyo, Japan, a leading pancreatic centre. PAR/PAD were performed by highly experienced surgeons with board certification initiated by the Japanese Society of Hepato-Biliary-Pancreatic Surgery. The tumours were anatomically reassessed as BRPC/LAPC according to National Comprehensive Cancer Network 2024 guidelines^10^. The cohort was categorically divided into two distinct groups to assess the impact of the timing of surgical interventions: patients undergoing surgery before 2016 (2010–2015), the former interval, and patients undergoing surgery after 2016 (2016–2021), the latter interval. This study was approved by the Institutional Review Board of the Cancer Institute Hospital (registration number 2020-GA-1330), and the requirement for written informed consent was waived.

### Surgical strategy

All surgical procedures were conducted using standardized techniques, as previously described^11–15^. Arterial resection for the hepatic artery, celiac axis and splenic artery during pancreatoduodenectomy (PD) was conducted from 2016 if deemed necessary to achieve R0 resection. Resection of the splenic artery during PD was performed for patients with pancreatic body cancer contacting with or invading the splenic artery. The simplest approach for hepatic artery resections involves removing a short segment of hepatic artery around the root of the gastroduodenal artery, followed by a direct end-to-end anastomosis between the common hepatic artery and the proper hepatic artery. For cases involving a longer defect, the artery, the middle colic artery or the splenic artery^11^ were transposed. In cases of pancreatic cancer in the body/tail of the pancreas involving the celiac axis, distal pancreatectomy with celiac axis resection (DP-CAR) was selected rather than PAD, referred to as level 3 dissection^14^. Even when the tumour contacted with >180 degrees of the superior mesenteric artery, PAD was the first choice whenever possible with preparing PAR^13,14^. During DP-CAR, the left gastric artery was reconstructed^15^ and a jejunal serosal patch covering the pancreatic stump has been performed since 2017^16^.

Para-aortic lymph nodes were dissected as a sample and sent as frozen sections^17^. When a metastasis was confirmed at the examination, curative resection was not standardized and depended on the surgeon’s discretion.

### Perioperative therapy

For BRPC, the treatment strategy shifted from upfront surgery to neoadjuvant chemotherapy (NAC) starting in 2015, basically using four courses of GNP^18–20^. For LAPC, the treatment strategy has been established since 2014, where a modified FOLFIRINOX or GNP regimen is selected based on patient age, general condition and family history^21^. Surgical indications for LAPC after induction chemotherapy were based on carbohydrate antigen 19-9 (CA19–9) concentration, tumour shrinkage at imaging, disease control for over 8 months and favourable patient condition according to the modified Glasgow prognostic score^21^. Adjuvant chemotherapy shifted from gemcitabine to S-1 (tegafur-gimeracil-oteracil potassium) in March 2013^13,18,22^. The duration of adjuvant chemotherapy was 6 months. No preoperative radiotherapy was performed.

### Definitions

The eighth edition of the AJCC TNM staging system was used^23^. Pathological residual tumour status was defined by resection margin status using the 0 mm rule. Postoperative complications were graded using the Clavien–Dindo classiﬁcation^24^. Postoperative pancreatic fistula (POPF), delayed gastric emptying and postpancreatectomy haemorrhage were deﬁned according to the International Study Group^25–27^.

### Recurrence patterns

Contrast-enhanced CT was performed every 3 months for 2 years after resection, and thereafter every 6 months until 5 years after resection. CA19-9 and carcinoembryonic antigen (CEA) levels were checked monthly during adjuvant therapy and every 3 months after that. When a lesion which was suspected of recurrence was detected, fludeoxyglucose-18 (FDG)-PET was performed. Early recurrence was defined as recurrence within 12 months after resection for PDAC. Local recurrence was defined as local tissue at the primary tumour, remnant pancreas or regional lymph nodes^28^.

### Statistical analysis

Categorical variables were compared using the Fisher’s exact test and continuous variables were compared using the Mann–Whitney *U* test. Overall survival (OS) was calculated from the date of starting treatment, including preoperative chemotherapy, to the date of death from any cause. Recurrence-free survival (RFS) was calculated from the date of pancreatectomy to the date of recurrence. Survival curves were constructed using the Kaplan–Meier method and compared using the log-rank test. The Cox proportional hazards regression model with backward elimination with a *P* value of 0.05 was used to identify factors independently associated with OS and RFS. To detect independent predictors for each specific site of recurrence, logistic regression was performed with multivariable analysis using factors significantly associated in univariable analysis. All statistical evaluations were performed using the SPSS 23 software package (SPSS Japan Inc., Tokyo, Japan). All tests were two-sided, and *P* < 0.05 were considered as statistically significant.

## Results

### Patient characteristics

A total of 584 patients were diagnosed as BRPC with arterial involvement (*n* = 213) or LAPC (*n* = 371) from 2010 to 2021. Among 584 patients, 203 patients underwent pancreatectomy with curative intent and were included in this study (*Fig. S1*). There were 79 patients in the former interval and 124 patients in the latter interval. In the former interval, 24 patients underwent PAR and 55 underwent PAD; in the latter interval, 52 underwent PAR and 72 underwent PAD. Following PAR, 41 patients underwent end-to-end anastomosis, while the remaining 35 did not undergo revascularization (*Tables 1* and *2*). The use of preoperative chemotherapy increased from the former interval to the latter interval (26.6% (*n* = 21) *versus* 95.2% (*n* = 118), *P* < 0.001). The latter patients exhibited lower pT (*P* = 0.002), pN (*P* = 0.002) and M (*P* = 0.015), as well as a lower incidence of R1 resection (*P* < 0.001). A similar proportion of patients received adjuvant chemotherapy between the former and latter intervals. Among patients who received adjuvant chemotherapy, half of the former patients (*n* = 32) received S-1 and 42.2% (*n* = 27) received gemcitabine, while most of the latter cases received S-1 (88.1% (*n* = 89)). Thirty-nine patients did not receive adjuvant chemotherapy due to early recurrence after surgery (*n* = 17), frailness (*n* = 9), no wish to receive (*n* = 7), postoperative morbidity rate (*n* = 3) or long duration of preoperative chemotherapy (*n* = 3).

**Table 1:** Patient characteristics

	All	Former period*	Latter period†	
	(*n* = 203)	(*n* = 79)	(*n* = 124)	*P*
Age (years), median (i.q.r.)	67 (59–72)	66 (58–71)	68 (59–73)	0.449
**Sex**				0.775
Male	95 (46.8)	38 (48.1)	57 (46.0)	
Female	108 (53.2)	41 (51.9)	67 (54.0)	
BMI, median (i.q.r.)	21.6 (19.5–23.4)	21.9 (19.5–23.3)	21.6 (19.4–23.5)	0.880
**ASA-PS**				0.601
I/II	187 (92.1)	74 (93.7)	113 (91.1)	
III/IV	16 (1.8)	5 (6.3)	11 (5.4)	
**Location of the tumour**				0.615
Head	131 (64.5)	50 (63.3)	81 (65.3)	
Body	64 (31.5)	27 (34.2)	37 (29.8)	
Tail	8 (3.9)	2 (2.5)	6 (4.8)	
**Resectability at diagnosis**				0.068
BRPC	164 (80.8)	69 (87.3)	95 (77.0)	
LAPC	39 (19.2)	10 (12.7)	29 (23.0)	
Portal vein contact on image	168 (82.8)	71 (89.9)	97 (78.2)	0.037
Serum CA19-9 level at diagnosis (U/ml), median (i.q.r.)	244 (29–975)	278 (42–1049)	216 (23–972)	0.407
Preoperative serum CA19-9 level (U/ml), median (i.q.r.)	30 (9–174)	121 (25–639)	18 (6–59)	<0.001
Use of preoperative chemotherapy	139 (68.5)	21 (26.6)	118 (95.2)	<0.001
**Regimen of preoperative chemotherapy**				<0.001
Gemcitabine plus nab-paclitaxel	118 (58.1)	11 (13.9)	107 (86.3)	
FOLFIRINOX	12 (5.9)	2 (2.5)	10 (8.1)	
Others	9 (4.4)	8 (10.2)	1 (0.8)	
**Type of pancreatectomy**				0.668
PD	133 (65.5)	53 (67.1)	80 (64.5)	
DP	65 (32.0)	25 (31.6)	40 (32.3)	
TP	5 (2.5)	1 (1.3)	4 (3.2)	
**Type of pancreatectomy and artery resection**				0.287
PD with hepatic artery	12 (5.9)	1 (1.3)	11 (8.9)	
PD with splenic artery	5 (2.5)	0	5 (4.0)	
PD with CA	4 (2.0)	0	4 (2.0)	
DP with CA	53 (26.1)	22 (27.8)	31 (15.3)	
TP with CA	2 (1.0)	1 (1.3)	1 (0.5)	
Concomitant portal vein resection	135 (66.5)	55 (69.6)	80 (64.5)	0.542

Values are *n* (%) unless otherwise stated. BRPC, borderline resectable pancreatic cancer; LAPC, locally advanced pancreatic cancer. *From 2010 to 2015. †From 2016 to 2021. BMI, body mass index; ASA-PS, American Society of Anesthesiologists physical status; CA19-9, carbohydrate antigen 19-9; PD, pancreaticoduodenectomy; DP, distal pancreatectomy; TP, total pancreatectomy; CA, celiac axis.

**Table 2:** Pathologic characteristics

	All	Former period*	Latter period†	
	(*n* = 203)	(*n* = 79)	(*n* = 124)	*P*
**Tumour differentiation**‡				0.015
Well	39 (19.2)	20 (25.3)	19 (15.3)	
Moderate	116 (57.2)	34 (43.0)	82 (66.1)	
Poor	46 (22.7)	24 (30.4)	22 (17.7)	
Others§	2 (0.9)	1 (1.3)	1 (0.8)	
**pT category**‡				0.002
T1	32 (15.8)	5 (6.3)	27 (21.8)	
T2	111 (54.7)	42 (43.0)	69 (55.6)	
T3	48 (23.6)	28 (35.4)	20 (16.1)	
T4	12 (5.9)	4 (5.1)	8 (6.5)	
**pN category**‡				0.002
N0	72 (35.5)	18 (22.8)	54 (43.5)	
N1	85 (41.9)	35 (44.3)	50 (37.1)	
N2	46 (22.6)	26 (32.9)	20 (13.7)	
**pM category**‡				0.015
M0	183 (90.1)	66 (83.5)	117 (94.4)	
M1¶	20 (9.9)	13 (32.9)	7 (13.7)	
**Lavage cytology**				0.051
Negative	189 (93.1)	70 (88.6)	119 (94.4)	
Positive	14 (6.9)	9 (11.4)	5 (5.6)	
**Residual tumour status**‡				<0.001
R0	172 (84.7)	56 (70.1)	116 (93.5)	
R1	31 (15.3)	23 (29.1)	8 (6.5)	

Values are *n* (%). *From 2010 to 2015. †From 2016 to 2021. ‡According to the AJCC TNM staging system, 8th edition^23^. §One with adenosquamous carcinoma and the other with acinar cell carcinoma. pT, pathological T; pN, pathological N; pM, pathological M. ¶The sites of metastasis were the para-aortic lymph nodes in 16 patients, the liver in three patients, and a para-aortic lymph node and peritoneum in a patient; R0, no residual tumour; R1, microscopic residual tumour.

Among patients undergoing PAR, compared with the former patients, the latter patients showed comparable tumour characteristics at diagnosis, but received preoperative chemotherapy more than the former patients (*Table S1*). Compared with the former patients, the latter patients had significantly lower preoperative serum CA19-9 levels and showed a tendency to have lower pT, pN and M categories as well as lower incidences of positive cytology and R1 resection (*Table S1*).

Compared with patients undergoing PAD, those undergoing PAR had a higher proportion of advanced disease, indicated by a higher proportion of LAPC, portal vein contact on image, portal vein resection and pT4 category (*Tables S2*).

Forty-five patients experienced some form of major morbidity rate (Clavien–Dindo classification grade Ⅲa or higher) (*Table 3*). Among patients undergoing PAD, there was only one 90-day death due to liver failure (*Table S3*). There were no fatalities among patients undergoing PAR (*Table S3*).

**Table 3:** Perioperative outcomes

	All	Former period*	Latter period†	
	(*n* = 203)	(*n* = 79)	(*n* = 124)	*P*
EBL (ml), median (i.q.r.)	600 (363–935)	580 (400–890)	610 (420–945)	0.515
Operating time (min), median (i.q.r.)	526 (450–622)	519 (447–603)	532 (450–532)	0.353
Blood transfusion	16 (7.9)	9 (11.4)	7 (5.6)	0.182
Major morbidity rate	46 (22.7)	24 (30.4)	22 (17.7)	0.040
POPF grade B/C§	33 (16.3)	20 (25.3)	13 (10.5)	0.006
PPH grade B/C§	9 (4.4)	3 (3.8)	6 (4.8)	0.182
DGE grade B/C§	36 (17.7)	20 (25.3)	16 (12.9)	0.037
Diarrhoea	41 (20.2)	14 (17.7)	27 (21.8)	0.591
Respiratory	6 (3.0)	1 (1.3)	5 (4.0)	0.408
Cardiac	1 (0.5)	1 (1.3)	0	0.389
Liver failure	3 (1.5)	2 (2.5)	1 (0.8)	0.561
Deep vien thrombosis/pulmonary embolism	2 (1.0)	0	2 (1.6)	0.522
PVT	1 (0.5)	1 (1.3)	0	0.389
SSI	27 (13.3)	15 (19.0)	12 (9.7)	0.088
Reoperation on	8 (3.9)	5 (6.3)	3 (2.4)	0.266
ICU admission	6 (3.0)	0	6 (4.8)	0.084
Hospital stay (days), median (i.q.r.)	27 (19–37)	34 (23–41)	24 (17–33)	0.002
Readmission	21 (10.3)	8 (10.1)	13 (10.5)	>0.999
90-day mortality rate	1 (0.5)	0	1 (0.8)	>0.999
Use of adjuvant chemotherapy	164 (80.8)	64 (81.0)	101 (81.5)	>0.999
**Regimen of adjuvant chemotherapy**				<0.001
S-1	120 (59.1)	32 (40.5)	89 (71.8)	
Gemcitabine	29 (14.3)	27 (34.2)	2 (1.6)	
Others	15 (7.4)	5 (6.3)	10 (8.1)	

Values are *n* (%) unless otherwise stated. *From 2010 through 2015. †From 2016 through 2021. EBL, estimated blood loss. POPF, postoperative pancreatic fistula; §According to International Study Group^25–27^. PPH, postpancreatectomy haemorrhage; DGE, delayed gastric emptying; PVT, portal vein thrombosis; SSI, surgical site infection; ICU, intensive care unit; S-1, tegafur-gimeracil-oteracil potassium.

Among patients undergoing PAR, the major morbidity rate and POPF significantly decreased over time. Delayed gastric emptying, diarrhoea requiring medication and duration of hospital stay showed a tendency to decrease over time (*Table S3*).

Compared with patients undergoing PAD, those undergoing PAR showed higher incidences of grade B/C POPF and diarrhoea requiring medication, and longer duration of hospital stay than those undergoing PAD (*Table S4*).

### Long-term survival

**Fig. 1:**
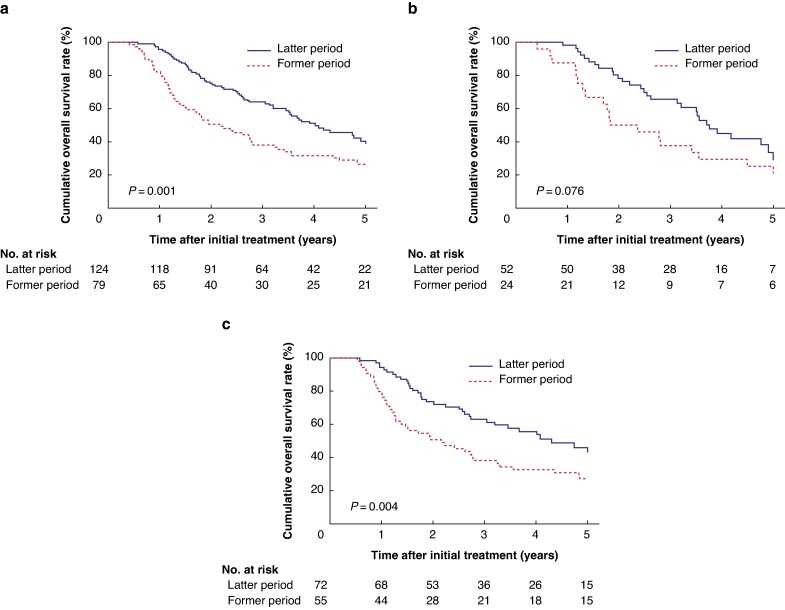
Kaplan–Meier survival curves of overall survival for patients with pancreatic cancer with arterial involvement

The median follow-up interval was 33.2 (range: 5.0–121.8) months. The cumulative 3- and 5-year OS rates were 53.7% and 35.3% respectively, with a median survival time (MST) of 41.0 months. The cumulative 3- and 5-year RFS rates were 25.6% and 18.5% respectively, with an MST of 14.1 months. Compared with the former patients, the latter had significantly better OS (5-year OS rate, 26.6% *versus* 40.6%; MST, 26.0 *versus* 48.2 months; *P*  *=* 0.001) (*Fig. 1a*). Meanwhile, RFS was comparable between the former and latter intervals (5-year RFS rate, 17.7% *versus* 17.6%; MST, 11.2 *versus* 16.3 months; *P*  *=* 0.264) (*Fig. S2*a). Local RFS was similar between the former and latter intervals (5-year local RFS rate, 23.6% *versus* 25.6%; MST, 21.3 *versus* 30.1 months; *P* = 0.142).

**a** Among all patients undergoing pancreatectomy, compared with patients in the former interval (from 2010 to 2015), those in the latter interval (from 2016 to 2021) had significantly better overall survival (median survival time (MST), 26.6 *versus* 48.2 months; *P* = 0.001). **b** Among patients undergoing pancreatectomy with arterial resection, compared with patients in the former interval, those in the latter interval had better overall survival (MST, 22.0 *versus* 45.1 months; *P* = 0.076). **c** Among patients undergoing pancreatectomy with arterial divestment, compared with patients in the former interval, those in the latter interval had significantly better overall survival (MST, 26.0 *versus* 51.7 months; *P* = 0.004).

Multivariable analysis for OS identified that a lower preoperative CA19-9 level, absence of portal vein contact on image, pN0, negative lavage cytology, use of adjuvant chemotherapy and surgery in the latter interval were independent favourable prognostic factors (*Table 4*).

**Table 4:** Univariable and multivariable analyses for overall survival in patients with arterial involvement

		Univariable analysis				Multivariable analysis	
	*n*	MST (months)	3-year survival rate (%)	5-year survival rate (%)	*P*	HR (95% c.i.)	*P*
**Age (years)**					0.534		
≤67	107	44.2	57.4	37.5			
>67	96	33.4	49.6	33.3			
**Sex**					0.603		
Male	95	36.6	51.0	35.1			
Female	108	42.1	56.2	35.4			
**ASA-PS**					0.177		
I/II	187	39.6	52.1	33.6			
III/IV	16	82.0	61.9	61.9			
**Location of the tumour**					0.796		
Head of pancreas	131	36.6	50.5	35.3			
Body of pancreas	64	42.7	59.7	34.4			
Tail of pancreas	8	–	60.0	–			
**Resectability at diagnosis**					0.070		
BRPC	164	36.6	50.0	34.4			
LAPC	39	57.2	69.1	43.0			
**Portal vein contact on image**					0.002		0.004
Absent	35	78.7	78.0	63.6			
Present	168	33.4	48.7	39.6		2.23 (1.29,3.86)	
**Serum CA19-9 level at diagnosis (U/ml)**					0.019		0.005
≤300	112	52.4	61.3	43.0			
>300	91	31.4	44.6	26.5		1.69 (1.17,2.44)	
**Preoperative serum CA19-9 level (U/ml)**					0.007		0.308
≤300	162	44.5	59.5	39.1			
>300	41	21.4	31.0	20.7		0.40 (0.73,2.65)	
**Use of preoperative chemotherapy**					0.001		0.180
No	64	19.4	34.4	26.2		1.23 (0.73,2.06)	
Yes	139	47.0	62.6	38.5			
**Type of pancreatectomy**					0.249		
PD	133	32.8	48.1	33.3			
DP/TP	70	47.0	64.5	38.9			
**PAD/PAR**					0.580		
PAD	127	39.0	52.2	37.5			
PAR	76	42.1	56.2	31.5			
**Concomitant portal vein resection**					0.004		0.204
Absent	68	58.9	69.5	47.8			
Present	135	32.5	45.9	29.2		1.34 (0.85,2.11)	
pT category*					0.736		
pT1/pT2/T3	191	41.0	53.5	35.7			
pT4	12	38.5	58.3	31.3			
pN category*					<0.001		
pN0	72	58.2	71.4	47.4		1.41 (0.91,2.20)	0.128
pN1	85	42.1	56.6	37.7		4.34 (2.67,7.05)	<0.001
pN2	46	18.4	21.7	12.9			
M category*					0.034		0.870
pM0	183	42.3	56.5	36.6			
pM1†	20	21.8	30.0	24.0		1.05 (0.60,1.82)	
**Lavage cytology**					0.002		0.010
Negative	189	42.7	56.7	36.9			
Positive	14	20.5	14.3	14.3		2.28 (1.22,4.29)	
Residual tumour status*					<0.001		
R0	172	42.8	57.7	38.8			
R1	31	20.5	31.9	17.7			
**Use of adjuvant chemotherapy**					0.012		<0.001
No	154	42.7	56.8	39.1			
Yes	49	26.7	43.9	23.2		2.37 (1.56,3.60)	
**Operative period**					0.001		0.010
Latter period‡	124	48.2	64.1	40.6			
Former period§	79	26.0	38.0	26.6		1.60 (1.12,2.29)	

BRPC, borderline resectable pancreatic cancer; LAPC, locally advanced pancreatic cancer; MST, median survival time; HR, hazard ratio; c.i., confidence interval; ASA-PS, American Society of Anesthesiologists physical status; CA19-9, carbohydrate antigen 19-9; PD, pancreatoduodenectomy; DP, distal pancreatectomy; TP, total pancreatectomy; PAD, pancreatectomy with arterial divestment; PAR, pancreatectomy with arterial resection; pT, pathological T. *According to the AJCC TNM staging system, 8th edition^23^; pN, pathological N; pM, pathological M. †The sites of metastasis were the para-aortic lymph nodes in 16 patients, the liver in three patients, and a para-aortic lymph node and peritoneum in a patient; R0, no residual tumour; R1, microscopic residual tumour. ‡From 2016 through 2021. §From 2010 through 2015.

Among patients undergoing PAR, OS prolonged over time when comparing the former and latter intervals (5-year OS rate, 25.0% *versus* 33.4%; MST, 22.0 *versus* 45.1 months; *P =* 0.076) (*Fig. 1b*), whereas RFS was comparable (5-year RFS rate, 8.3% *versus* 9.5%; MST, 8.3% *versus* 14.8%; *P =* 0.203) (*Fig. S2b*).

Among patients undergoing PAD, compared with the former patients, the latter patients showed significantly better OS than the former (5-year OS rate, 27.3% *versus* 45.9%; MST, 26.0 *versus* 51.7 months; *P =* 0.004) (*Fig. 1c*), while RFS was equivalent between the former and latter intervals (5-year RFS rate, 12.5% *versus* 25.0%; MST, 12.5 *versus* 17.6 months; *P =* 0.419) (*Fig. S2c*).

Patients undergoing PAD and those undergoing PAR showed comparable OS (5-year OS rate, 37.5% *versus* 31.5%; MST, 39.0 *versus* 42.1 months; *P =* 0.580), RFS (5-year RFS rate, 23.0% *versus* 10.1%; MST, 15.1 *versus* 11.9 months; *P =* 0.071) and local RFS (5-year local RFS rate, 28.2% *versus* 20.9%; MST, 26.4 *versus* 25.9 months; *P* = 0.479).

In a subgroup analysis for patients without M1 disease (*n* = 183), compared with the former patients, the latter had significantly better OS (5-year OS rate, 27.3% *versus* 42.1%; MST, 26.0 *versus* 49.0 months; *P*  *=* 0.003) (*Fig. S3a*), even among patients undergoing PAR (*P =* 0.101) (*Fig. S3b*) and those undergoing PAD (*P*  *=* 0.011) (*Fig. S3c*). Meanwhile, RFS was comparable between the former and latter intervals (5-year RFS rate, 19.7% *versus* 17.4%; MST, 11.9 *versus* 16.3 months; *P*  *=* 0.583) (*Fig. S4a*), even among patients undergoing PAR (*Fig. S4b*) and those undergoing PAD (*Fig. S4c*).

### Recurrence

In the entire cohort, early recurrence occurred in 112 patients. Compared with the former patients, the latter experienced less early recurrence (54.4% (*n* = 43) *versus* 38.7% (*n* = 48), *P =* 0.031) (*Table S5*). Patients undergoing PAR and PAD showed comparable incidences of early recurrence (*P =* 0.307). A multivariable analysis revealed that being male, a high serum CA19-9 level at diagnosis, pN2 and absence of adjuvant chemotherapy were independent predictors of early recurrence (*Table 5*).

**Table 5:** Multivariable regression model: independent predictors of early recurrence

	Odds ratio (95% c.i.)	*P*
**Sex**		0.038
Male	1.97 (1.04,3.74)	
Female		
**Serum CA19-9 level at diagnosis (ml)**		0.004
<300		
≥300	2.54 (1.34,4.84)	
pN category*		
N0		
N1	1.36 (0.67,2.78)	0.395
N2	6.19 (2.54,15.10)	<0.001
**Use of adjuvant chemotherapy**		0.026
Yes		
No	2.58 (1.12,5.92)	

*According to the AJCC TNM staging system, 8th edition^23^. pN, pathological N; c.i., confidence interval; CA19-9, carbohydrate antigen 19-9.

Local recurrence occurred in 32 patients, distant in 91, local and distant in 34 (*Table S5*). The distribution of these patterns was equivalent between the former and latter intervals (*P* = 0.601), and between PAR and PAD (*P* = 0.441).

**Fig. 2:**
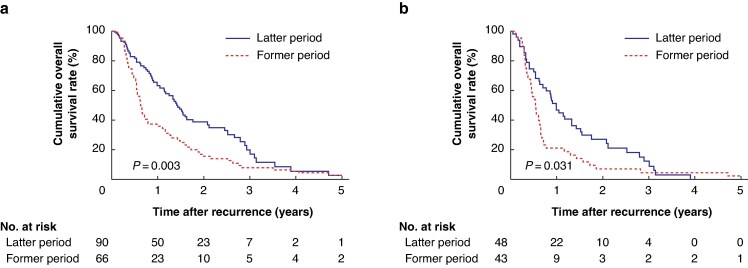
Kaplan–Meier survival curves of postrecurrence survival following pancreatectomy for pancreatic cancer with arterial involvement

Among all 156 patients with recurrence, the cumulative 1- and 3-year postrecurrence survival (PRS) rates were 51.1% and 14.3% respectively, and MST for PRS was 13.2 months. Compared with the former patients, the latter had significantly better PRS (*P* = 0.003) (*Fig. 2a*), even among patients with early recurrence (*P* = 0.031) (*Fig. 2b*).

**a** Among all patients with recurrence following pancreatectomy for pancreatic cancer with arterial involvement, compared with patients in the former interval (from 2010 to 2015), those in the latter interval (from 2016 to 2021) had significantly better postrecurrence survival (median survival time (MST), 7.5 *versus* 17.1 months; *P* = 0.003). **b** Among patients with early recurrence (less than 12 months after surgery), compared with patients in the former interval, those in the latter interval had significantly better overall MST (6.5 *versus* 11.9 months; *P =* 0.031).

## Discussion

The present study demonstrated that short- and long-term outcomes of patients with pancreatic cancer with arterial involvement have improved with the introduction of modern multidisciplinary treatment in recent decades. The results showed that PAR can be safely performed with acceptable morbidity and mortality rates and long-term outcomes for patients with pancreatic cancer with arterial involvement in specialized high-volume centres.

The oncologic outcomes following PAR for pancreatic cancer were historically dismal. In 2020, Bachellier *et al*. reported an MST of 13.7 months after PAR for 118 patients with pancreatic cancer, three-quarters of whom received preoperative chemotherapy^6^. In 2022, Loos *et al*. demonstrated that MST was 17.7 months after PAR for LAPC patients, 48.7% of whom received preoperative chemotherapy^7^. The present decadal analysis revealed a significant improvement in oncologic outcomes for patients with arterial involvement over time. Although preoperative chemotherapy did not emerge as a prognostic factor for OS in multivariable analysis, independent prognostic factors of preoperative serum CA19-9 levels, *N* category and incidence of positive lavage cytology were lower in the latter interval, which were attributable to the increased adoption of modern preoperative chemotherapy during the latter interval. Thus, the introduction of preoperative chemotherapy comprising modern multiagent regimens may have contributed to the improvement of oncologic outcomes after PAR for BRPC/LAPC.

PAR remains a challenging surgical procedure due to severe postoperative morbidity rates. Recent reports from high-volume centres of pancreatic surgery also demonstrated high major morbidity (24.6–54.1%) and mortality rates (4.2–13.5%) following PAR^6,7,29^, but 13.5–30% of patients underwent the invasive procedure of superior mesenteric artery resection, while 15.3–61.0% of patients underwent total pancreatectomy, eliminating the risk of POPF. The present study indicated a relatively low major morbidity rate (22.2%) and low mortality rate (0.5%), even in patients undergoing PAR, but it did not include patients with superior mesenteric artery resection.

A multidisciplinary perioperative care bundle has been implemented from 2015^30^. The preservation and reconstruction of the left gastric artery during DP-CAR resulted in a low incidence of ischaemia^15,16,31^. The application of a jejunal serosal patch to cover the pancreatic stump after DP-CAR effectively reduced the occurrence of POPF^15,16,31–33^. The implementation of multidisciplinary perioperative care played a significant role in reducing morbidity and mortality rates among patients undergoing PAR^34^.

Recent studies reported a recurrence rate of 82–88% after resection for BRPC/LAPC^35,36^, and reported that local recurrence was the most prevalent (27.5%), followed by the liver (14.8%) and the lung (9.5%)^35^, which are almost identical to these data. Regrettably, local RFS did not improve over time, despite a significant improvement in the R0 resection rate with the introduction of modern preoperative chemotherapy. Verbeke *et al*. highlighted the difficulty in pathological evaluation of resection margin status following NAC, indicating that microscopic tumour foci may persist in extrapancreatic areas and extend over considerable distances after NAC, resulting in residual microscopic disease despite achieving ypR0 resection^37^.

Patients in the latter interval experienced fewer early recurrences, probably due to higher preoperative chemotherapy treatment (which controlled potential micrometastasis, selected patients who may benefit from radical resection^38^ and caused a lower pN status) and adjuvant chemotherapy^22^. This study also revealed that survival after recurrence prolonged in the latter interval even among patients with early recurrence, which would also have been attributable to modern multiagent chemotherapy.

When comparing postoperative outcomes between PAR and PAD, PAD was generally the first choice for PC patients with arterial involvement in this centre, and patients undergoing PAR had more aggressive disease with a higher proportion of LAPC. Even though such selection bias and differences in the basic characteristics existed, both PAD and PAR were safely performed and patient selection for PAR was feasible. The proportion of diarrhoea was significantly higher after PAR than after PAD, probably because the nerve plexus around the superior mesenteric artery tends to be more severely affected among patients undergoing PAR for their more aggressive disease.

There are several limitations in this study, including its retrospective and single-centre study design without measuring quality of life, the long study interval, the heterogeneity in patient selection, preoperative/adjuvant chemotherapy regimen and technical reproducibility. In addition, early recurrence was difficult to confirm especially in patients having PAD/PAR with or without portal vein resection. However, when a lesion suspected of local recurrence was detected, and it was difficult to confirm whether it was recurrence or not, FDG PET was performed.

Within the context of modern multidisciplinary treatment, radical resection including arterial resection should be justified for patients with pancreatic cancer with arterial involvement at expert centres, considering the acceptable perioperative risk and potential for prolonged survival.

## Supplementary Material

zraf026_Supplementary_Data

## Data Availability

The data sets generated and analysed during the present study are not publicly available, as consent was not provided by the participants to share their data with third parties.
