# Supplementary material for: Arterial resection and divestment in pancreatic cancer surgery in the era of multidisciplinary treatment: decadal comparative study
Source: BJS Open. 2025 Apr 17;9(2):zraf026. doi: 10.1093/bjsopen/zraf026 (PMC12005265; doi:10.1093/bjsopen/zraf026)
Supplement: zraf026_Supplementary_Data [file zraf026_supplementary_data.docx]

**The Feasibility and Effectiveness of Pancreatic Cancer Surgery with the Arterial Resection/Divestment in the Era of Multidisciplinary Treatment: A Decadal Comparative Study**

Yuki Hirose ^1,2^, Atsushi Oba ^1,^*, Yosuke Inoue^1^, Aya Maekawa^1^, Kosuke Kobayashi^1^, Kojiro Omiya^1^, Atsushi Takahashi^1^, Yoshihiro Ono^1^, Takafumi Sato^1^, Hiromichi Ito^1^, Takafumi Mie^1^, Takashi Sasaki^1^, Masato Ozaka^1^, Naoki Sasahira^1^, Toshifumi Wakai^1^, Yu Takahashi^1,^*

* Shared supervisors.

^1^Division of Hepatobiliary and Pancreatic Surgery, Cancer Institute Hospital, Japanese Foundation for Cancer Research, Tokyo, Japan.

^2^Division of Digestive and General Surgery, Niigata University Graduate School of Medical and Dental Sciences, Niigata, Japan.

^3^Department of Hepato-Biliary-Pancreatic Medicine, Cancer Institute Hospital, Japanese Foundation for Cancer Research, Tokyo, Japan.

**Corresponding author.**

Yu Takahashi and Atsushi Oba, Division of Hepatobiliary and Pancreatic Surgery, Cancer Institute Hospital, Japanese Foundation for Cancer Research, 3-8-31 Ariake, Koto-Ku, Tokyo, 135-8550, Japan.

Phone: 813-3520—0111; Fax: 813-3520-0141

E-mail: yu.takahashi@jfcr.or.jp and [atsushi.oba@jfcr.or.jp](mailto:atsushi.oba@jfcr.or.jp)

**ORCID: https://orcid.org/0000-0002-7315-0830**

**Supplementary Materials - Index**

| **Supplementary Methods** |  |
| --- | --- |
| a multidisciplinary perioperative care bundle | *page 3* |
| **Supplementary Figures and Tables** |  |
| Supplementary table S1. Basic characteristics of patients with BRPC/LAPC who underwent PAR | *page 4* |
| Supplementary table S2. Basic characteristics of patients with BRPC/LAPC who underwent PAR and PAD | *page 6* |
| Supplementary table S3. Perioperative outcomes of patients with BRPC/LAPC with arterial involvement undergoing PAR/PAD: comparison between former and latter period | *Page 8* |
| Supplementary table S4. Perioperative outcomes of patients with BRPC/LAPC with arterial involvement undergoing PAR/PAD | *Page 9* |
| Supplementary figure S1. Flow chart of patients with BRPC/LAPC with arterial involvement | *page 10* |
| Supplementary figure S2. Kaplan-Meier survival curves of recurrence-free survival for pancreatic cancer patients with arterial involvement | *page 11* |
| Supplementary figure S3. Kaplan-Meier survival curves of overall survival for pancreatic cancer patients with arterial involvement without distant metastasis | *page 12* |
| Supplementary figure S4. Kaplan-Meier survival curves of recurrence-free survival for pancreatic cancer patients with arterial involvement without distant metastasis | *page 13* |
| Supplementary table S5. Recurrence patterns | *Page 14* |

**Supplementary Methods**

For elderly patients undergoing PD from 2015 through 2016, and for all patients undergoing PD from 2017, a multidisciplinary perioperative care bundle was implemented and include oral hygiene, physical fitness assessment, rehabilitation, and nutritional support to reduce postoperative morbidity (AU: you should move this paragraph in Supplementary material).

**Supplementary Figures and Tables**

| **Table S1 Basic characteristics of patients with BRPC/LAPC who underwent PAR** | | | | |
| --- | --- | --- | --- | --- |
|  | **All** | **Former period**^*^ | **Latter period**^†^ |  |
| **Variable** | **(n = 76)** | **(n = 24)** | **(n = 52)** | **P** |
| Age^‡^ | 66 (56–72) | 65 (55–69) | 68 (57–73) | 0.178 |
| Sex |  |  |  | 0.088 |
| Male | 36 (47.4%) | 15 (62.5%) | 21 (40.4%) |  |
| Female | 40 (52.6%) | 9 (37.5%) | 31 (59.6%) |  |
| BMI^‡^ | 21.6 (19.9–23.6) | 22.4 (20.8–23.5) | 21.5 (19.4–24.3) | 0.219 |
| ASA-PS |  |  |  | 0.648 |
| I/II | 71 (93.4%) | 22 (91.7%) | 49 (94.2%) |  |
| III/IV | 5 (6.7%) | 2 (8.3%) | 3 (5.8%) |  |
| Location of the tumour |  |  |  | 0.004 |
| Head | 23 (30.3%) | 2 (8.3%) | 21 (40.4%) |  |
| Body | 51 (67.1%) | 20 (83.4%) | 31 (59.6%) |  |
| Tail | 2 (2.6%) | 2 (8.3%) | 0 (0%) |  |
| Resectability at diagnosis |  |  |  | >0.999 |
| BRPC | 48 (63.2%) | 15 (62.5%) | 33 (63.5%) |  |
| LAPC | 28 (36.8%) | 9 (37.5%) | 19 (36.5%) |  |
| Portal vein contact on image | 53 (69.7%) | 19 (79.2%) | 34 (65.4%) | 0.288 |
| Serum CA19-9 level at diagnosis^‡^ | 405 (30–1185) | 441 (66–1617) | 280 (24–1169) | 0.325 |
| Preoperative serum CA19-9 level (U/mL) ^‡^ | 29 (9–212) | 90 (17–650) | 18 (7–102) | 0.004 |
| Use of preoperative chemotherapy | 57 (75.0%) | 9 (37.5%) | 48 (92.3%) | <0.001 |
| Regimen of preoperative chemotherapy |  |  |  | <0.001 |
| Gemcitabine plus nab-paclitaxel | 46 (60.5%) | 4 (16.7%) | 42 (80.8%) |  |
| FOLFIRINOX | 7 (9.2%) | 1 (4.2%) | 6 (11.5%) |  |
| Others | 4 (5.3%) | 4 (16.7%) | 0 (0%) |  |
| Type of pancreatectomy |  |  |  | 0.008 |
| PD | 21 (27.6%) | 1 (4.2%) | 20 (38.5%) |  |
| DP | 53 (69.7%) | 22 (91.6%) | 31 (59.6%) |  |
| TP | 2 (2.6%) | 1 (4.2%) | 1 (1.9%) |  |
| Concomitant portal vein resection | 37 (48.7%) | 8 (33.3%) | 29 (55.8%) | 0.087 |
| Tumour differentiation^§^ |  |  |  | 0.107 |
| Well | 9 (11.8%) | 4 (16.7%) | 5 (9.6%) |  |
| Moderate | 42 (55.3%) | 9 (37.5%) | 33 (63.5%) |  |
| Poor | 25 (32.9%) | 11 (45.8%) | 14 (26.9%) |  |

| **Table S1 (continued)** | | | | |
| --- | --- | --- | --- | --- |
|  | **All** | **Former period**^*^ | **Latter period**^†^ |  |
| **Variable** | **(n = 76)** | **(n = 24)** | **(n = 52)** | **P** |
| pT category^§^ |  |  |  | 0.096 |
| T1 | 12 (15.8%) | 1 (4.2%) | 11 (21.2%) |  |
| T2 | 32 (42.1%) | 9 (37.4%) | 23 (44.2%) |  |
| T3 | 20 (26.3%) | 10 (41.7%) | 10 (19.2%) |  |
| T4 | 12 (15.8%) | 4 (16.7%) | 8 (15.4%) |  |
| pN category^§^ |  |  |  | 0.151 |
| N0 | 24 (31.6%) | 4 (16.7%) | 20 (38.5%) |  |
| N1 | 38 (50.0%) | 14 (58.3%) | 24 (46.1%) |  |
| N2 | 14 (18.4%) | 6 (25.0%) | 8 (15.4%) |  |
| pM category^§^ |  |  |  | 0.015 |
| M0 | 66 (86.8%) | 18 (75.0%) | 48 (92.3%) |  |
| M1 | 10 (13.2%) | 6 (25.0%) | 4 (7.7%) |  |
| Lavage cytology |  |  |  | 0.051 |
| Negative | 70 (92.1%) | 20 (83.3%) | 50 (96.2%) |  |
| Positive | 6 (7.9%) | 4 (16.7%) | 2 (3.8%) |  |
| Residual tumour status^§^ |  |  |  | <0.001 |
| R0 | 67 (88.2%) | 19 (79.2%) | 48 (92.3%) |  |
| R1 | 9 (11.8%) | 5 (20.8%) | 4 (7.7%) |  |
| Use of adjuvant chemotherapy | 59 (77.6%) | 19 (79.1%) | 40 (76.9%) | 0.183 |
| Regimen of adjuvant chemotherapy |  |  |  | <0.001 |
| S-1 | 47 (61.8%) | 9 (37.5%) | 38 (73.1%) |  |
| Gemcitabine | 9 (11.8%) | 8 (33.3%) | 1 (1.9%) |  |
| Others | 3 (3.9%) | 2 (8.3%) | 1 (1.9%) |  |
| Values are n (%) unless otherwise stated. BRPC, borderline resectable pancreatic cancer; LAPC, locally advanced pancreatic cancer; PAR indicates pancreatectomy with arterial resection; ^*^From 2010 through 2015. ^†^From 2016 through 2021. ^‡^Data were expressed as median (IQR). BMI, body mass index; ASA-PS, American Society of Anesthesiologists physical status; CA19-9, carbohydrate antigen 19-9; PD, pancreaticoduodenectomy; DP, distal pancreatectomy; TP, total pancreatectomy; ^§^According to the AJCC TNM staging system, 8th edition^23^. pT, pathological T; pN, pathological N; pM, pathological M; R0, no residual tumour; R1, microscopic residual tumour; S-1, tegafur-gimeracil-oteracil potassium. | | | | |

| **Table S2 Basic characteristics of patients with BRPC/LAPC who underwent PAR and PAD** | | | | |
| --- | --- | --- | --- | --- |
|  | **All** | **PAR** | **PAD** |  |
| **Variable** | **(n = 203)** | **(n = 76)** | **(n = 127)** | **P** |
| Age^*^ | 67 (59–72) | 66 (56–72) | 67 (60–72) | 0.200 |
| Sex |  |  |  | >0.999 |
| Male | 95 (46.8%) | 36 (47.4%) | 59(46.5%) |  |
| Female | 108 (53.2%) | 40 (52.6%) | 68 (53.5%) |  |
| BMI^*^ | 21.6 (19.5–23.4) | 21.6 (19.9–23.6) | 21.8 (19.1–23.3) | 0.293 |
| ASA-PS |  |  |  | 0.789 |
| I/II | 187 (92.1%) | 71 (93.4%) | 116 (91.3%) |  |
| III/IV | 16 (1.8%) | 5 (6.7%) | 11 (8.7%) |  |
| Location of the tumour |  |  |  | <0.001 |
| Head | 131 (64.5%) | 23 (30.3%) | 108 (85.0%) |  |
| Body | 64 (31.5%) | 51 (67.1%) | 13 (10.2%) |  |
| Tail | 8 (3.9%) | 2 (2.6%) | 6 (4.7%) |  |
| Resectability at diagnosis |  |  |  | <0.001 |
| BRPC | 164 (80.8%) | 48 (63.2%) | 116 (91.3%) |  |
| LAPC | 39 (19.2%) | 28 (36.8%) | 11 (8.7%) |  |
| Portal vein contact on image | 168 (82.8%) | 53 (69.7%) | 115 (90.6%) | <0.001 |
| Serum CA19-9 level at diagnosis^*^ | 244 (29–975) | 405 (30–1185) | 183 (25–912) | 0.285 |
| Preoperative serum CA19-9 level (U/mL)^*^ | 30 (9–174) | 29 (9–212) | 33 (9–154) | 0.937 |
| Use of preoperative chemotherapy | 139 (68.5%) | 57 (75.0%) | 82 (64.6%) | 0.160 |
| Regimen of preoperative chemotherapy |  |  |  | 0.420 |
| Gemcitabine plus nab-paclitaxel | 118 (58.1%) | 46 (60.5%) | 72 (56.8%) |  |
| FOLFIRINOX | 12 (5.9%) | 7 (9.2%) | 5 (3.9%) |  |
| Others | 9 (4.4%) | 4 (5.3%) | 5 (3.9%) |  |
| Type of pancreatectomy |  |  |  | <0.001 |
| PD | 133 (65.5%) | 21 (27.6%) | 112 (88.2%) |  |
| DP | 65 (32.0%) | 53 (69.7%) | 12 (9.4%) |  |
| TP | 5 (2.5%) | 2 (2.6%) | 3 (2.3%) |  |
| Concomitant portal vein resection | 135 (66.5%) | 37 (48.7%) | 98 (77.2%) | <0.001 |
| Tumour differentiation^†^ |  |  |  | 0.016 |
| Well | 39 (19.2%) | 9 (11.8%) | 30 (23.6%) |  |
| Moderate | 116 (57.2%) | 42 (55.3%) | 74 (58.3%) |  |
| Poor | 46 (22.7%) | 25 (32.9%) | 21 (16.5%) |  |
| Others^‡^ | 2 (0.9%) | 0 | 2 (1.6%) |  |

| **Table S2 (continued)** | | | | |
| --- | --- | --- | --- | --- |
|  | **All** | **PAR** | **PAD** |  |
| **Variable** | **(n = 76)** | **(n = 76)** | **(n = 127)** | **P** |
| pT category^†^ |  |  |  | <0.001 |
| T1 | 32 (15.8%) | 12 (15.8%) | 20 (15.8%) |  |
| T2 | 111 (54.7%) | 32 (42.1%) | 79 (62.2%) |  |
| T3 | 48 (23.6%) | 20 (26.3%) | 28 (22.0%) |  |
| T4 | 12(5.9%) | 12 (15.8%) | 0 |  |
| pN category^†^ |  |  |  | 0.183 |
| N0 | 72 (35.5%) | 24 (31.6%) | 48 (37.8%) |  |
| N1 | 85 (41.9%) | 38 (50.0%) | 47 (37.0%) |  |
| N2 | 46 (22.6%) | 14 (18.4%) | 32 (25.2%) |  |
| pM category^†^ |  |  |  | 0.233 |
| M0 | 183 (90.1%) | 66 (86.8%) | 117 (92.1%) |  |
| M1 | 20 (9.9%) | 10 (13.2%) | 10 (7.9%) |  |
| Lavage cytology |  |  |  | 0.776 |
| Negative | 189 (93.1%) | 70 (92.1%) | 119 (93.7%) |  |
| Positive | 14 (6.9%) | 6 (7.9%) | 8 (6.3%) |  |
| Residual tumour status^†^ |  |  |  | 0.321 |
| R0 | 172 (84.7%) | 67 (88.2%) | 105 (82.7%) |  |
| R1 | 31 (15.3%) | 9 (11.8%) | 22 (17.3%) |  |
| Use of adjuvant chemotherapy | 164 (80.8%) | 59 (77.6%) | 106 (83.5%) | 0.354 |
| Regimen of adjuvant chemotherapy |  |  |  | 0.331 |
| S-1 | 120 (59.1%) | 47 (61.8%) | 74 (58.3%) |  |
| Gemcitabine | 29 (14.3%) | 9 (11.8%) | 20 (15.8%) |  |
| Others | 15 (7.4%) | 3 (3.9%) | 12 (9.4%) |  |
| Values are n (%) unless otherwise stated. BRPC, borderline resectable pancreatic cancer; LAPC, locally advanced pancreatic cancer; PAR indicates pancreatectomy with arterial resection; PAD, pancreatectomy with arterial divestment. ^*^Data were expressed as median (IQR). BMI, body mass index; ASA-PS, American Society of Anesthesiologists physical status; CA19-9, carbohydrate antigen 19-9; PD, pancreaticoduodenectomy; DP, distal pancreatectomy; TP, total pancreatectomy; ^†^According to the AJCC TNM staging system, 8th edition^23^. ^‡^One with adenosquamous carcinoma and the other with acinar cell carcinoma. pT, pathological T; pN, pathological N; pM, pathological M; R0, no residual tumour; R1, microscopic residual tumour; S-1, tegafur-gimeracil-oteracil potassium. | | | | |

| **Table S3 Perioperative outcomes of patients with BRPC/LAPC with arterial involvement undergoing PAR/PAD: comparison between former and latter period** | | | | | | | |
| --- | --- | --- | --- | --- | --- | --- | --- |
|  |  | **PAR** | |  | **PAD** | |  |
|  | **All** | **Former period**^*^ | **Latter period**^†^ |  | **Former period**^*^ | **Latter period**^†^ |  |
| **Variable** | **(n = 203)** | **(n = 24)** | **(n = 52)** | **P** | **(n = 55)** | **(n = 72)** | **P** |
| EBL (mL)^‡^ | 600 (363–935) | 590 (363–935) | 680(476–1155) | 0.288 | 667 (432–975) | 580 (389–856) | 0.859 |
| Operation time (min)^‡^ | 526 (450–622) | 468 (361–554) | 544 (450–659) | 0.014 | 546 (485–615) | 526 (434–653) | 0.551 |
| Blood transfusion | 16 (7.9%) | 1 (4.2%) | 3 (5.8%) | >0.999 | 8 (14.5%) | 4 (5.6%) | 0.125 |
| Major morbidity | 46 (22.7%) | 12 (50.0%) | 10 (19.2%) | 0.013 | 12 (21.8%) | 12 (16.7%) | 0.499 |
| POPF grade B/C^§^ | 33 (16.3%) | 13 (54.2%) | 5 (9.6%) | <0.001 | 7 (20.0%) | 8 (11.1%) | 0.789 |
| PPH grade B/C^§^ | 9 (4.4%) | 1 (4.2%) | 4 (7.7%) | >0.999 | 2 (3.6%) | 2 (2.8%) | >0.999 |
| DGE grade B/C^§^ | 36 (17.7%) | 8 (33.3%) | 8 (15.4%) | 0.128 | 12 (21.8%) | 8 (11.1%) | 0.140 |
| Diarrhea | 41 (20.2%) | 13 (54.2%) | 18 (34.6%) | 0.135 | 1 (1.8%) | 9 (12.5%) | 0.049 |
| Respiratory | 6 (3.0%) | 0 | 0 |  | 1 (1.8%) | 5 (6.9%) | 0.233 |
| Cardiac | 1 (0.5%) | 1 (4.2%) | 0 | 0.316 | 0 | 0 |  |
| Liver failure | 3 (1.5%) | 0 | 0 |  | 2 (3.6%) | 1 (1.4%) | 0.578 |
| DVT/PE | 2 (1.0%) | 0 | 2 (3.8%) | >0.999 | 0 | 0 |  |
| PVT | 1 (0.5%) | 0 | 0 |  | 1 (1.8%) | 0 | 0.433 |
| SSI | 27 (13.3%) | 3 (12.5%) | 2 (3.8%) | 0.318 | 12 (21.8%) | 10 (13.9%) | 0.344 |
| Reoperation | 8 (3.9%) | 2 (8.3%) | 2 (3.8%) | 0.587 | 3 (5.5%) | 1 (1.4%) | 0.315 |
| ICU admission | 6 (3.0%) | 0 | 1 (1.9%) | >0.999 | 0 | 5 (6.9%) | 0.082 |
| Hospital stay (days)^‡^ | 27 (19–37) | 35 (21–53) | 30 (21–38) | 0.081 | 28 (23–38) | 20 (17–30) | 0.001 |
| Readmission | 21 (10.3%) | 3 (12.5%) | 8 (15.4%) | >0.999 | 5 (9.1%) | 5 (6.9%) | 0.519 |
| 90-day Mortality | 1 (0.5%) | 0 | 0 |  | 0 | 1 (1.4%) | >0.999 |
| Values are n (%) unless otherwise stated. BRPC, borderline resectable pancreatic cancer; LAPC, locally advanced pancreatic cancer; PAR indicates pancreatectomy with arterial resection; PAD, pancreatectomy with arterial divestment; ^*^From 2010 through 2015. ^†^From 2016 through 2021. EBL, estimated blood loss; ^‡^Data were expressed as median (IQR). POPF, postoperative pancreatic fistula; ^§^According to International Study Group^25-27^. PPH, postpancreatectomy hemorrhage; DGE, delayed gastric emptying; DVT, deep vein thrombosis; PE, pulmonary embolism; PVT, portal vein thrombosis; SSI, Surgical Site Infection; ICU, Intensive Care Unit. | | | | | | | |

| **Table S4 Perioperative outcomes of patients with BRPC/LAPC with arterial involvement undergoing PAR/PAD** | | | | |
| --- | --- | --- | --- | --- |
|  | **All** | **PAR** | **PAD** |  |
| **Variable** | **(n = 203)** | **(n = 76)** | **(n = 127)** | **P** |
| EBL (mL)^*^ | 600 (363–935) | 667 (432–975) | 735 (400–860) | 0.178 |
| Operation time (min)^*^ | 526 (450–622) | 530 (408–622) | 537 (459–624) | 0.325 |
| Blood transfusion | 16 (7.9%) | 4 (5.3%) | 12 (9.4%) | 0.421 |
| Major morbidity | 46 (22.7%) | 22 (28.9%) | 24 (18.9%) | 0.119 |
| POPF grade B/C^†^ | 33 (16.3%) | 18 (23.7%) | 15 (11.8%) | 0.051 |
| PPH grade B/C^†^ | 9 (4.4%) | 5 (6.6%) | 4 (3.1%) | 0.299 |
| DGE grade B/C^†^ | 36 (17.7%) | 16 (21.1%) | 20 (15.8%) | 0.349 |
| Diarrhea | 41 (20.2%) | 31 (40.8%) | 10 (7.9%) | <0.001 |
| Respiratory | 6 (3.0%) | 0 | 6 (4.7%) | 0.086 |
| Cardiac | 1 (0.5%) | 1 (1.3%) | 0 | 0.374 |
| Liver failure | 3 (1.5%) | 0 | 3 (2.4%) | 0.294 |
| DVT/PE | 2 (1.0%) | 2 (2.6%) | 0 | 0.139 |
| PVT | 1 (0.5%) | 0 | 1 (0.8%) | >0.999 |
| SSI | 27 (13.3%) | 5 (6.6%) | 22 (17.3%) | 0.033 |
| Reoperation | 8 (3.9%) | 4 (5.3%) | 4 (3.1%) | 0.476 |
| ICU admission | 6 (3.0%) | 1 (1.3%) | 5 (3.9%) | 0.408 |
| Hospital stay (days)^*^ | 27 (19–37) | 31 (21–41) | 25 (18–36) | 0.033 |
| Readmission | 21 (10.3%) | 11 (14.5%) | 10 (7.9%) | 0.236 |
| 90-day Mortality | 1 (0.5%) | 0 | 1 (0.8%) | >0.999 |
| Values are n (%) unless otherwise stated. BRPC, borderline resectable pancreatic cancer; LAPC, locally advanced pancreatic cancer; PAR indicates pancreatectomy with arterial resection; PAD, pancreatectomy with arterial divestment; EBL, estimated blood loss; ^*^Data were expressed as median (IQR). POPF, postoperative pancreatic fistula; ^†^According to International Study Group^25-27^. PPH, postpancreatectomy hemorrhage; DGE, delayed gastric emptying; DVT, deep vein thrombosis; PE, pulmonary embolism; PVT, portal vein thrombosis; SSI, Surgical Site Infection; ICU, Intensive Care Unit. | | | | |

**Fig. S1** **Flow chart of patients with BRPC/LAPC with arterial involvement**

**
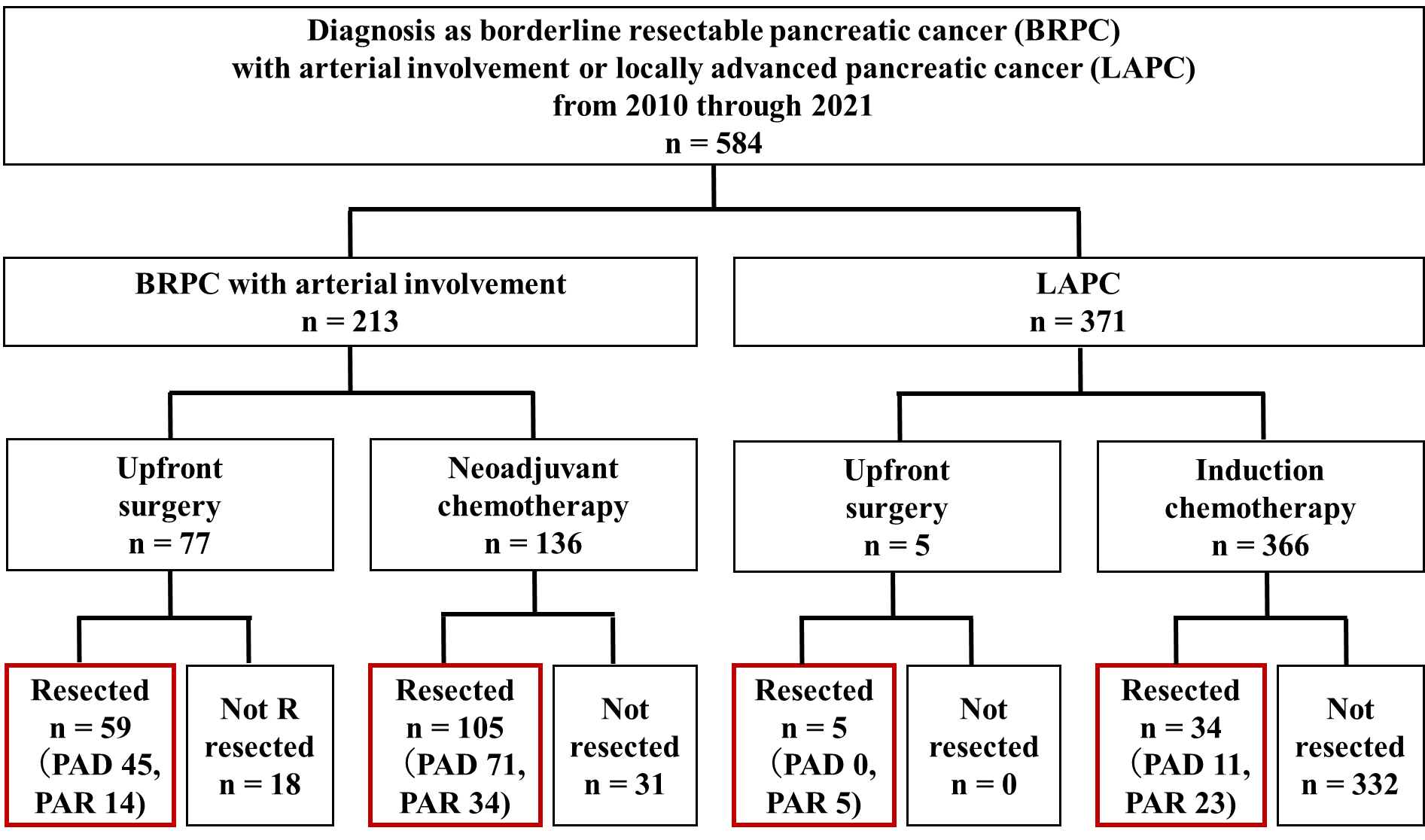
**

**Fig. S2** **Kaplan-Meier survival curves of recurrence-free survival for pancreatic cancer patients with arterial involvement**

**a** Among all patients undergoing pancreatectomy, there was no significant difference in recurrence-free survival between patients in the former (from 2010 through 2015) and latter period (from 2016 through 2021) (median survival time [MST], 11.2 vs. 16.3 months; P *=* 0.264). **b** Among patients undergoing pancreatectomy with arterial resection, there was no significant difference in recurrence-free survival between patients in the former and latter period (MST, 8.3 vs. 14.8 months; P *=* 0.203). **c** Among patients undergoing pancreatectomy with arterial divestment, there was no significant difference in recurrence-free survival between patients in the former and latter period (12.5 vs. 17.6 months; P *=* 0.419).


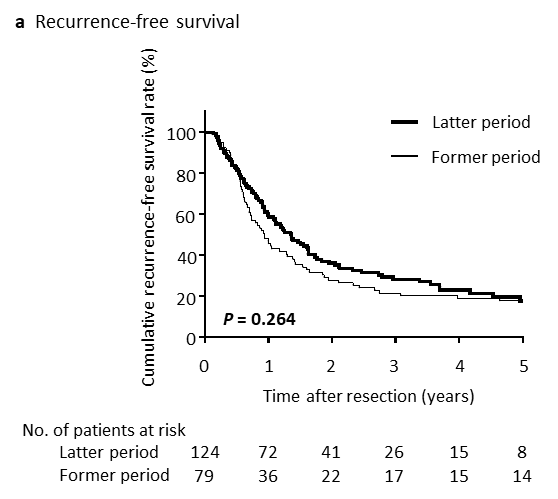

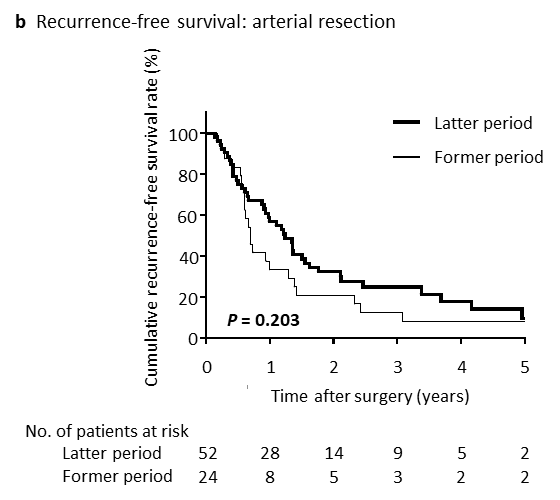

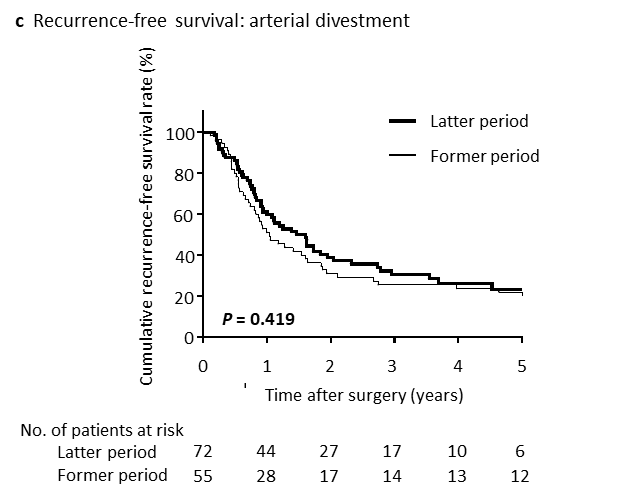


**Fig. S3** **Kaplan-Meier survival curves of overall survival for pancreatic cancer patients with arterial involvement without distant metastasis**

**a** Among all patients without distant metastasis who underwent pancreatectomy (n = 183), compared with patients in the former period (from 2010 through 2015), those in the latter period (from 2016 through 2021) had significantly better overall survival (OS) (median survival time [MST], 26.0 vs. 49.0 months; P = 0.003). **b** Among patients without distant metastasis who underwent pancreatectomy with arterial resection, compared with the former patients, the latter showed better OS (MST, 21.3 vs. 44.5 months; P = 0.101). **c** Among patients without distant metastasis who underwent pancreatectomy with arterial divestment, the latter patients had significantly better OS (MST, 26.7 vs. 56.9 months; P = 0.011).


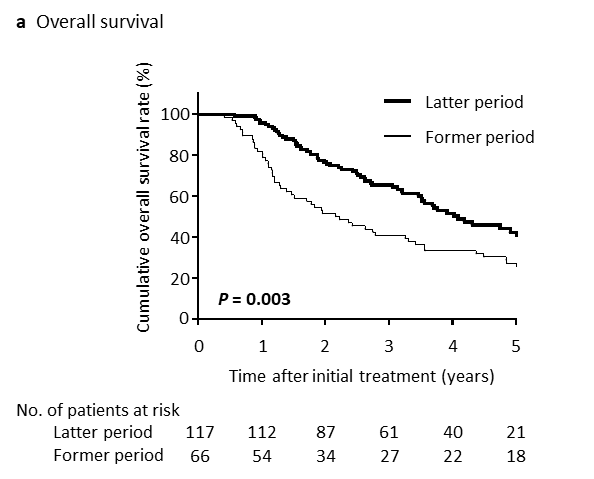

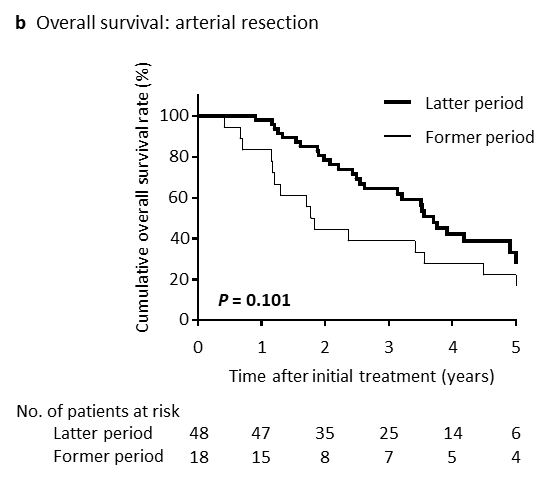

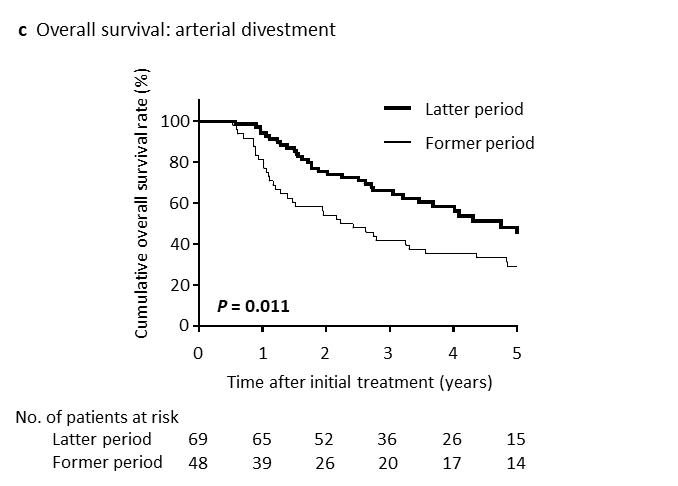


**Fig. S4** **Kaplan-Meier survival curves of recurrence-free survival for pancreatic cancer patients with arterial involvement without distant metastasis**

**a** Among all patients without distant metastasis who underwent pancreatectomy (n = 183), recurrence-free survival was comparable between the former (from 2010 through 2015) and the latter periods (from 2016 through 2021) (median survival time [MST], 11.9 vs. 16.3 months; P = 0.583). **b** Among patients without distant metastasis who underwent pancreatectomy with arterial resection, there was no significant difference in recurrence-free survival between patients in the former and latter period (MST, 8.3 vs. 16.2 months; P = 0.579). **c** Among patients undergoing pancreatectomy with arterial divestment, there was no significant difference in recurrence-free survival between patients in the former and latter period (MST, 12.6 vs. 19.5 months; P = 0.548).


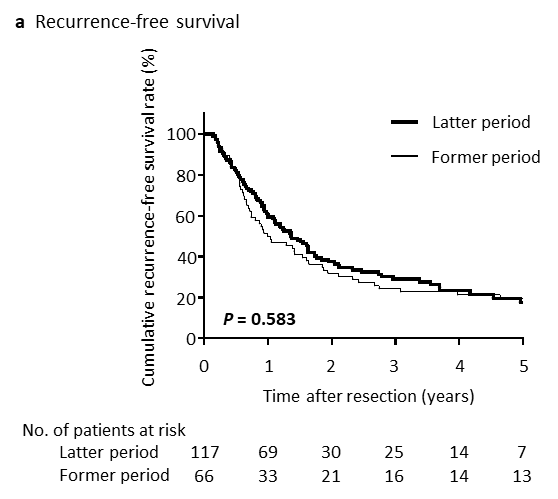

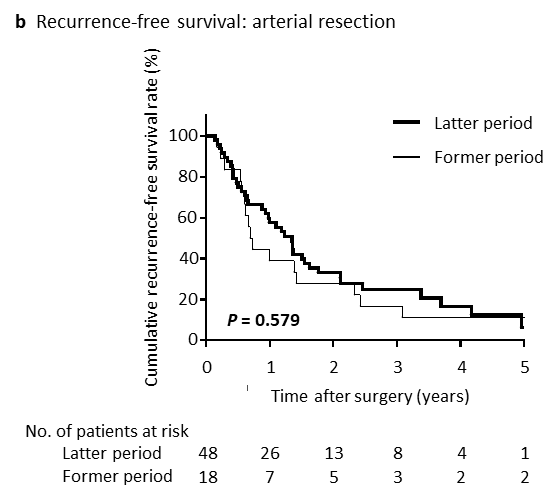

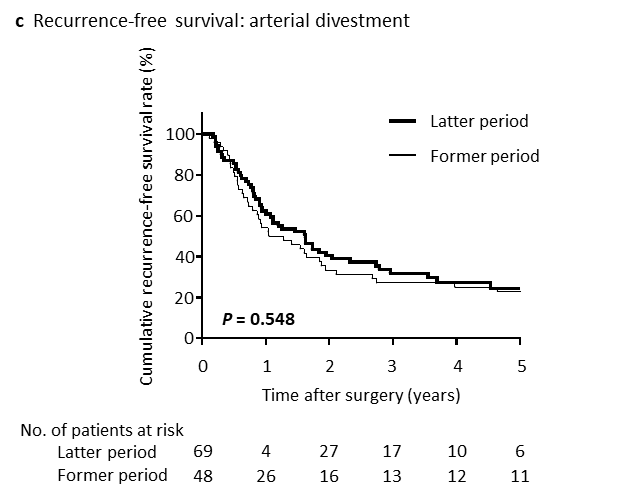


| **Table S5 Recurrence patterns** | | | | | | | | |
| --- | --- | --- | --- | --- | --- | --- | --- | --- |
| **Variable** | **All** | **Former period**^*^ | **Latter period**^†^ | **P** |  | **PAR** | **PAD** | **P** |
| Timing of recurrence | 203 | 79 | 124 |  |  | 76 | 127 |  |
| Early recurrence (<12 months) | 112 (55.2%) | 43 (54.4%) | 48 (38.7%) | 0.031 |  | 38 (50.0%) | 53 (41.7%) | 0.307 |
| No / late recurrence (≥12 months) | 91 (44.8%) | 36 (45.6%) | 76 (61.3%) |  |  | 38 (50.0%) | 74 (58.3%) |  |
| Patterns of recurrence | 156 | 66 | 90 |  |  | 63 | 93 |  |
| Local recurrence | 32 (20.5%) | 15 (22.7%) | 17 (18.9%) | 0.601 |  | 12 (19.0%) | 20 (21.5%) | 0.441 |
| Distant recurrence | 90 (57.7%) | 35 (53.0%) | 55 (61.1%) |  |  | 40 (63.5%) | 50 (53.8%) |  |
| Local and distant recurrence | 34 (21.8%) | 16 (24.2%) | 18 (20.0%) |  |  | 11 (17.5%) | 23 (24.7%) |  |
| Initial site of recurrence | 156 | 66 | 90 |  |  | 63 | 93 |  |
| Local | 32 (20.5%) | 15 (22.7%) | 17 (18.9%) | 0.555 |  | 12 (19.0%) | 20 (21.5%) | 0.840 |
| Lung | 23 (14.7%) | 8 (12.1%) | 15 (16.7%) | 0.498 |  | 13 (20.6%) | 10 (10.8%) | 0.108 |
| Liver | 22 (14.1%) | 8 (12.1%) | 14 (15.6%) | 0.644 |  | 8 (12.7%) | 14 (15.1%) | 0.816 |
| Peritoneum | 17 (10.9%) | 6 (9.1%) | 11 (12.2%) | 0.610 |  | 8 (12.7%) | 9 (9.7%) | 0.606 |
| Lymph node | 10 (6.4%) | 2 (3.0%) | 8 (8.9%) | 0.192 |  | 4 (6.3%) | 6 (6.5%) | >0.999 |
| Others | 2 (1.3%) | 1 (1.5%) | 1 (1.1%) | >0.999 |  | 0 | 2 (2.2%) | 0.515 |
| Multiple site | 50 (32.1%) | 26 (39.4%) | 24 (26.7%) | 0.118 |  | 18 (28.6%) | 32 (34.4%) | 0.488 |
| Values are n (%). ^*^From 2010 through 2015. ^†^From 2016 through 2021. PAR indicates pancreatectomy with arterial resection; PAD, pancreatectomy with arterial divestment. | | | | | | | | |
